# Microbiome and climate: skin microbial diversity and community functions of *Polypedates megacephalus* (Anura: Rhacophoridae) associated with bioclimate

**DOI:** 10.1128/spectrum.02358-24

**Published:** 2025-03-05

**Authors:** Dan Sun, Yewei Liu, Shipeng Zhou, Madhava Meegaskumbura

**Affiliations:** 1 Guangxi Key Laboratory of Beibu Gulf Marine Biodiversity Conservation, College of Marine Sciences, Beibu Gulf University, Qinzhou, China; 2 Guangxi Key Laboratory for Forest Ecology and Conservation, College of Forestry, Guangxi University, Nanning, Guangxi, China; Brigham Young University, Provo, Utah, USA

**Keywords:** skin microbiota, amphibian, climatic factors, *Polypedates megacephalus*, conservation

## Abstract

The microbiome inhabiting animal skin plays a crucial role in host fitness by influencing both the composition and function of microbial communities. Environmental factors, including climate, significantly impact microbial diversity and the functional attributes of these communities. However, it remains unclear how specific climatic factors affect amphibian skin microbial composition, community function, and the relationship between these two aspects. Understanding these effects is particularly important because amphibians are poikilotherms and, thus, more susceptible to temperature fluctuations. Here, we investigated the skin microbiome of the rhacophorid tree frog *Polypedates megacephalus* across different climatic regimes using 16S rRNA gene sequencing. Skin swab samples were collected from nine populations of *P. megacephalus* adults in the Guangxi region, China. The majority of the core microbiota were found to belong to the genus *Pseudomonas*. Our findings indicate that microbial community diversity, composition, and function are associated with changes in climatic conditions. Specifically, the taxonomic and functional diversity of the skin microbiome increased in response to higher climate variability, particularly in temperature fluctuations. Additionally, the functional traits of microbial communities changed in parallel with shifts in community diversity and composition. The significant correlations of the functional redundancy index with climatic factors suggest that environmental filtering driven by climate change impacts microbial community functional stability. These results highlight the critical influence of climatic factors on amphibian skin microbiomes and offer new insights into how microbial composition and function contribute to host adaptation in varying environmental conditions.

**IMPORTANCE:**

This study is important in understanding the association between climate variability, microbial diversity, and host adaptation in amphibians, which are particularly vulnerable to environmental changes due to their poikilothermic nature. Amphibians rely on their skin microbiome for key functions like disease resistance, yet little is known about how climate fluctuations impact these microbial communities. By analyzing the microbiome of *Polypedates megacephalus* across different climatic regimes, our analysis reveals that warmer climates could reduce the microbial diversity and community functional redundancy, indicating the functional stability of skin microbiome could be susceptible to climate variability, particularly in hosts adapted to relatively cooler conditions. These findings highlight the potential ecological consequences of climate change and emphasize the need to integrate microbiome health into amphibian conservation strategies.

## INTRODUCTION

The microbiome inhabiting host skin plays a key role in host fitness by altering its community composition and function (1–3). Many studies have shown that changes in microbiome diversity or dominant bacterial communities have a negative impact on the health of human, wild, and domesticated animals (4–8). The characteristics of skin microbiota can be shaped by biotic factors (e.g., host-related traits) and abiotic factors (e.g., temperature and water quality) (9). In particular, climate-related parameters have been highlighted as one vital element for understanding ecology and evolution of host-associated microbiome (10).

Amphibians serve as the crucial components in both aquatic and terrestrial ecosystems, with their significance extending even to human health (11, 12). The exposed skin of amphibians, as the key component determining respiration, thermoregulation, osmoregulation, pigmentation, and protection from predators and pathogens, exhibits extremely sensitive to environmental variations (13). The skin microbial community compositions and interactions play an important role in the dynamics of infectious diseases (14, 15), such as chytridiomycosis, defined as the most destructive amphibian skin disease (16).

Several studies show the relationships between bioclimate and amphibian skin microbial community (10, 17–20); for example, skin microbiota exhibited more diversity in the colder and less stable temperature conditions compared with those in warm and less temperature variations (10). Nevertheless, Bacigalupe et al. (21) did not find a significant relationship between skin bacterial communities of the four-eyed frog (*Pleurodema thaul*) and climate factors. The different interpretations for the effects of ecological factors on the structure and compositions of skin bacterial diversity are dependent on different scale-based measurements (e.g., biogeographic gradients and host species) (22). Given the influence of host-specific and biogeographic area on host skin microbiome, it is necessary to perform an in-depth study of the structure and compositions of skin bacterial community under the context of various climatic conditions. Not only will this improve the understanding of how climate factors influence skin microbiota, but it can also predict the adaptation of amphibians to the rapid changes in climate.

Environmental conditions, including climate, not only affect microbial diversity but also have profound effects on the functional attributes of the microbial community (23, 24), while few studies have considered the functional attributes of amphibian skin microbial community under the context of climatic regimes (19). On the other hand, environmental changes are able to affect the relationships between microbial community composition and functional attributes due to the different degrees of influences of environmental filtering on microbial functional redundancy (25), where multiple distinct taxa have similar functions (26). However, much less known is about the effects of climatic variations on the relationships between amphibian skin microbial community and their functions.

*Polypedates megacephalus*, an Old-World tree frog (family Rhacophoridae), is widespread in southern China and Southeast Asia, occupying a broad niche space (27). Given the species’ wide geographic range and the significant impact of climate on animal microbiome, we hypothesized that the skin microbiota of *P. megacephalus* is closely linked to climatic factors. To test this, we sampled skin microbiota from populations inhabiting different sites along a latitudinal gradient in Guangxi, China, characterized by varying environmental conditions. We predicted that microbial community diversity and composition would be strongly influenced by climate-related factors and that the diversity and composition of microbial community functional attributes would show correlations (positive or negative) with climatic characteristics. Additionally, we anticipated that microbial community functional attributes and taxonomic diversity would exhibit similar responses to climatic variations, reflecting the influence of climate fluctuations on microbial functional redundancy.

## RESULTS

**Fig 1:**
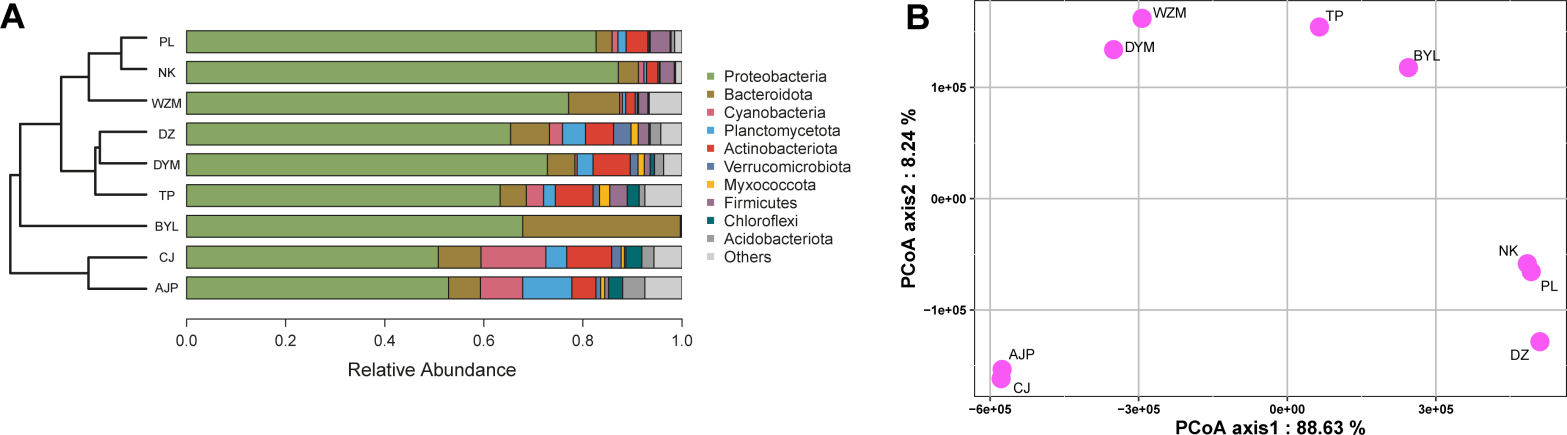
Unweighted pair group with arithmetic mean and stacked bar of skin bacteria at the phylum level. The composition of bacteria varied for *P. megacephalus* populations across sampling sites (**A**). The principal coordinate analysis (PCoA) scatterplots show the distribution of sampling sites based on Euclidean distance (**B**). The abbreviations of these geographic sites are as follows: AJP, Anjiangping; BYL, Biyun Lake; CJ, Cujiang; DYM, Dayao Mountain; DZ, Dongzhong; NK, Nakuan; PL, Pinglong; TP, Tianping; WZM, Wuzhi Mountain.

The taxonomic composition of operational taxonomic units (OTUs) on *P. megacephalus* frog skin bacteria predominately consisted of the phyla Proteobacteria, Bacteroidota, Actinobacteriota, Cyanobacteria, Planctomycetota, and Firmicutes, with relative abundances of 69.76%, 6.89%, 5.33%, 3.68%, 2.84%, and 2.47%, respectively. Hierarchical clustering of microbiome revealed that *P. megacephalus* skin had a site-specific microbial community structure (Fig. 1).

A total 56 OTUs were identified as a core bacterial community that dominated skin bacteriome of most frogs (Table 1). The core community exhibited 46% average relative abundance (minimum 8%, maximum 96%). The majority of core OTUs were identified as belonging to the genus *Pseudomonas*, which accounted for 10.20% of average relative abundance per frog. One individual with the lowest core community abundance (3%) was dominated by the members of the family Pasteurellaceae.

**TABLE 1:** Taxonomy of core microbiome present on 90% of *P. megacephalus* frogs sampled

OUT_ID	Taxonomy	Average relative abundance (%)
1, 408, 1207, 7097, 8613, 13995	p__Proteobacteria, c__Gammaproteobacteria, o__Pseudomonadales, f__Pseudomonadaceae, g__Pseudomonas	12.24
2, 94, 1075, 1174, 1201, 9030, 9732, 11586, 11850, 12242, 12717, 12839	p__Proteobacteria, c__Gammaproteobacteria, o__Gammaproteobacteria, f__Comamonadaceae	10.36
3	p__Proteobacteria, c__Gammaproteobacteria, o__Enterobacterales, f__Enterobacteriaceae, g__Cedecea	9.19
4	p__Proteobacteria, c__Gammaproteobacteria, o__Burkholderiales, f__Comamonadaceae, g__Comamonas	1.67
8	p__Proteobacteria, c__Gammaproteobacteria, o__Enterobacterales, f__Hafniaceae, g__Hafnia-Obesumbacterium	1.18
11	p__Proteobacteria, c__Gammaproteobacteria, o__Burkholderiales, f__Oxalobacteraceae, g__Janthinobacterium	0.90
14	p__Proteobacteria, c__Gammaproteobacteria, o__Xanthomonadales, f__Xanthomonadaceae, g__Stenotrophomonas	0.61
15	p__Firmicutes, c__Bacilli, o__Exiguobacterales, f__Exiguobacteraceae, Exiguobacterium	0.91
16, 36, 37	p__Proteobacteria; c__Alphaproteobacteria; o__Rhizobiales; f__Rhizobiaceae; g__Allorhizobium-Neorhizobium-Pararhizobium-Rhizobium	1.50
17	p__Proteobacteria, c__Gammaproteobacteria, o__Burkholderiales, f__Comamonadaceae, g__Pseudorhodoferax	1.30
19	p__Actinobacteriota, c__Actinobacteria, o__Micrococcales, f__Microbacteriaceae, g__Microbacterium	0.76
23, 121	p__Proteobacteria, c__Alphaproteobacteria, o__Rhizobiales, f__Devosiaceae, g__Devosia	0.49
26	p__Proteobacteria, c__Gammaproteobacteria, o__Burkholderiales, f__Oxalobacteraceae, g__Herbaspirillum	0.44
28	p__Proteobacteria, c__Alphaproteobacteria, o__Rhizobiales, f__Beijerinckiaceae, g__Bosea	0.30
31	p__Proteobacteria, c__Gammaproteobacteria, o__Pseudomonadales, f__Moraxellaceae, g__Enhydrobacter	0.22
48	p__Proteobacteria, c__Alphaproteobacteria, o__Rhizobiales, f__Xanthomonadaceae, g__Bradyrhizobium	0.39
52	p__Proteobacteria, c__Gammaproteobacteria, o__Pseudomonadales, f__Moraxellaceae, g__Acinetobacter	0.28
60	Proteobacteria, c__ Gammaproteobacteria, o__Burkholderiales, f__Oxalobacteraceae, g__Duganella	0.13
73	p__Proteobacteria, c__Gammaproteobacteria, o__Burkholderiales, f__Burkholderiaceae, Burkholderia-Caballeronia-Paraburkholderia	0.22
74	p__Actinobacteriota, c__Actinobacteria, o__Micrococcales, f__Micrococcaceae, g__Arthrobacter	0.12
83	p__Proteobacteria, c__Alphaproteobacteria, o__Rhizobiales, f__Beijerinckiaceae	0.18
92	p__Proteobacteria, c__Alphaproteobacteria, o__Sphingomonadales, f__Sphingomonadaceae, g__Hephaestia	0.14
100	p__Proteobacteria, c__Gammaproteobacteria, o__Enterobacterales, f__Erwiniaceae	0.14
102	p__Actinobacteriota, c__Actinobacteria, o__Micrococcales, f__Intrasporangiaceae	0.10
147	p__Proteobacteria, c__Alphaproteobacteria, o__Rhizobiales, f__Rhizobiaceae, g__Pseudochrobactrum	0.09
156	p__Actinobacteriota, c__Actinobacteria, o__Micrococcales, f__Microbacteriaceae, g__Leifsonia	0.09
402	p__Proteobacteria, c__Alphaproteobacteria, o__Sphingomonadales, f__Sphingomonadaceae, g__Novosphingobium	0.05
571, 808, 10525	p__Proteobacteria, c__Alphaproteobacteria, o__Sphingomonadales, f__Sphingomonadaceae, g__Sphingomonas	0.28
663	p__Actinobacteriota, c__Actinobacteria, o__Micrococcales, f__Microbacteriaceae, g__Galbitalea	0.35
727	p__Proteobacteria, c__Gammaproteobacteria, o__Burkholderiales, f__Oxalobacteraceae, g__Massilia	0.52
1628	p__Actinobacteriota, c__Actinobacteria, o__Micrococcales, f__Cellulomonadaceae, g__Cellulomonas	0.04
5256	p__Proteobacteria, c__Gammaproteobacteria, o__Enterobacterales, f__Enterobacteriaceae	0.13
10178	p__Proteobacteria, c__Gammaproteobacteria, o__Burkholderiales, f__Comamonadaceae, g__Acidovorax	0.28
13900	p___Proteobacteria, c__Gammaproteobacteria, o__Enterobacterales	0.51
13990	p___Proteobacteria, c__Gammaproteobacteria, o__Enterobacterales, f__Yersiniaceae	0.16

**Fig 2:**
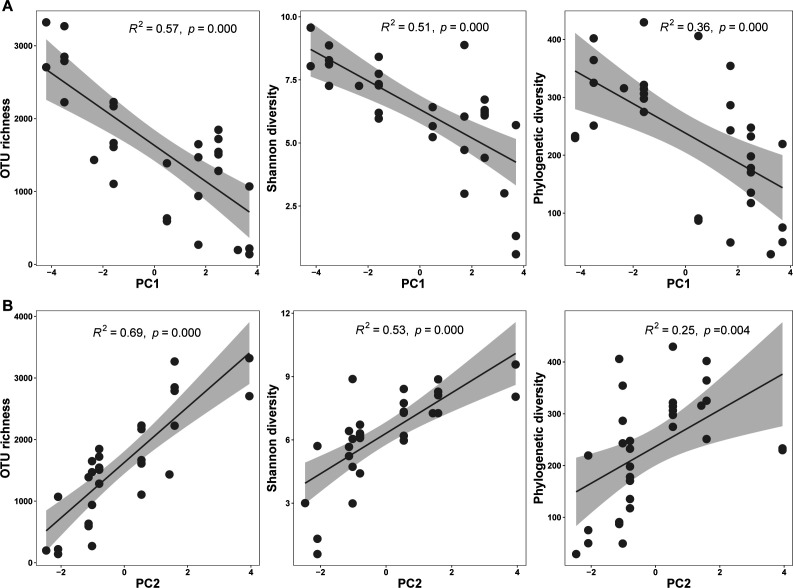
Linear regression plots between microbial alpha diversity with temperature-related factor (**A**) and precipitation-related factor (**B**). Gray shading represents 95% confidence interval.

Skin microbial alpha diversity indices had significant relationships with climatic factors (Mantel test: Pearson’s *r* = 0.162, *P* = 0.024; Fig. 2). The frogs exhibited increased skin microbial richness and phylogenetic diversity in colder or drier climate (Fig. S1). For instance, skin microbiota showed a decrease of alpha diversity with the minimum temperature of the coldest month (bio6) (richness: *R*^2^ = 0.526, *P* < 0.0001; Shannon: *R*^2^ = 0.469, *P* < 0.0001; phylogenetic diversity [PD]: *R*^2^ = 0.319, *P* = 0.001).

The microbial beta diversity had significant associations with both temperature factor (PC1; *F* = 3.17, *R*^2^ = 0.10, *P* < 0.0001) and precipitation factor (PC2; *F* = 2.01, *R*^2^ = 0.06, *P* = 0.006) (Fig. 2A and B), consistent with the result of the Mantel test (Pearson’s *r* = 0.234, *P* = 0.001).

**Fig 3:**
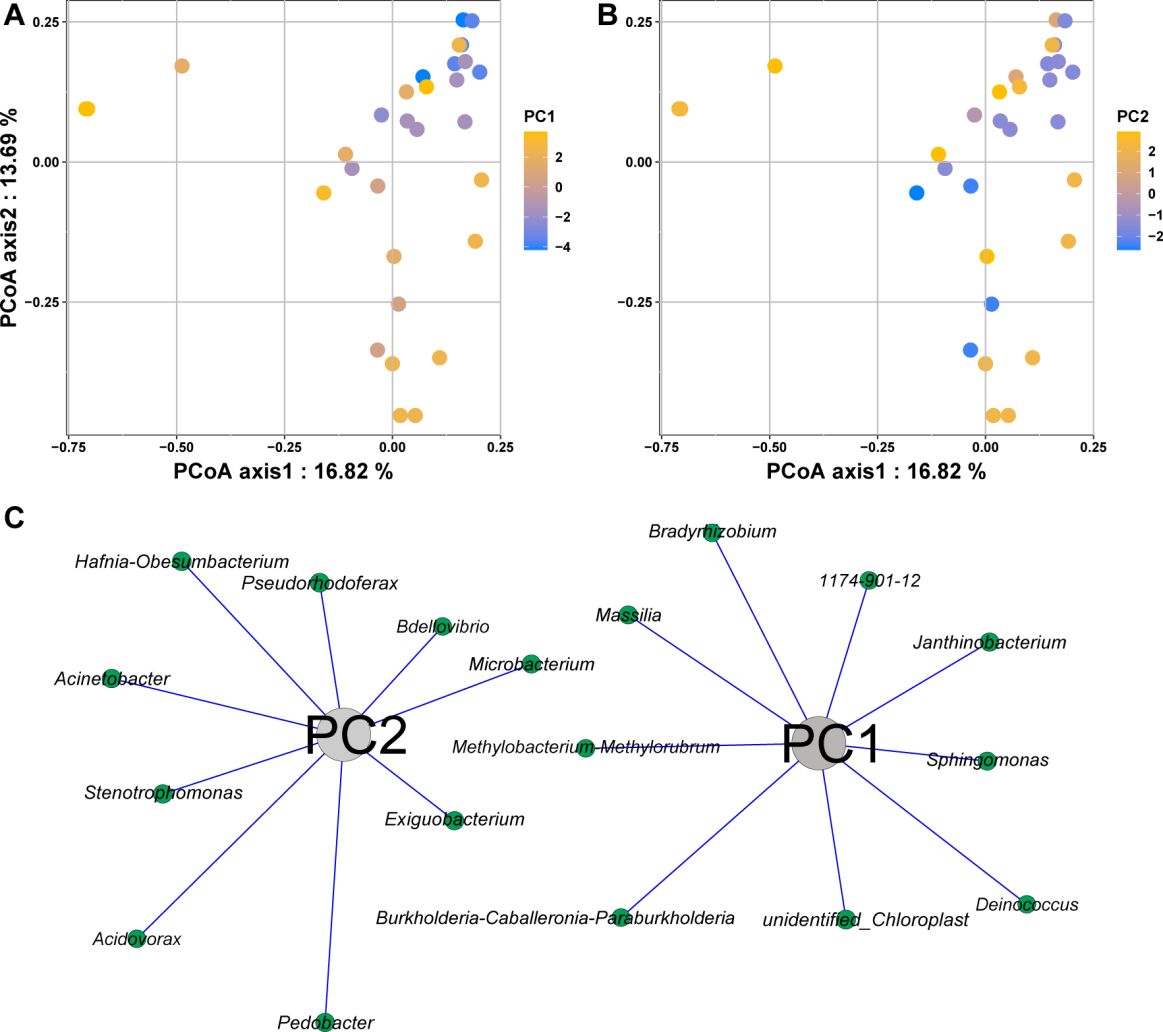
PCoA scatterplots show the variations in microbial community composition based on Bray–Curis distance and the correlations of bacterial genera with climatic indices. The variations in microbial community composition in the context of temperature-related factors (**A**) and precipitation-related factors (**B**). The bacterial genera have highly significant relationships with temperature-related factors (PC1) and precipitation-related factors (PC2) (Spearman’s *r* > |0.75|, *P* < 0.01) (**C**).

We found that the members of the Beijerinckiaceae, Xanthobacteraceae, Burkholderiaceae, Deinococcaceae, Oxalobacteraceae, Sphingomonadaceae, and unidentified_Chloroplast families correlated with the temperature factor, which increased with colder climate; the members of Comamonadaceae, Moraxellaceae, Bdellovibrionaceae, Exiguobacteraceae, Hafniaceae, Microbacteriaceae, Sphingobacteriaceae, and Xanthomonadaceae families correlated with the precipitation factor (Fig. 3C).

Most functional features were associated to metabolism (48.65% ± 0.019%). The top five functional categories in the Kyoto Encyclopedia of Genes and Genomes (KEGG) pathways (level 2 KEGG Ortholog [KO]) included membrane transport (12.81% ± 0.018%), amino acid metabolism (10.25% ± 0.006%), carbohydrate metabolism (9.75% ± 0.003%), replicate and repair (6.56% ± 0.003%), and energy metabolism (5.46% ± 0.005%).

**Fig 4:**
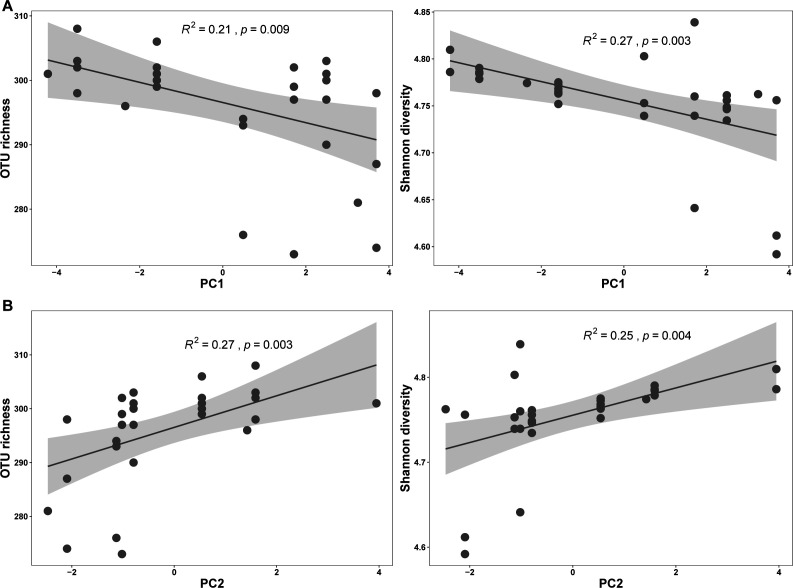
Linear regression plots between microbial functional diversity and temperature-related factor (**A**) and precipitation-related factor (**B**). Shading represents 95% CI.

The climatic factors significantly influenced the functional diversity of the skin microbiome of the tree frogs (Fig. 4), with the change tendency similar to microbial diversity under the context of PC1 and PC2 (Fig. 1).

The variations in skin microbial function compositions significantly correlated with bioclimatic variables in the Mantel test (Spearman’ *r* = 0.150, *P* = 0.031). This pattern was also found in the evaluation of the associations of functional dissimilarity with the temperature-related factor (*F* = 4.789, *R*^2^ = 0.142, *P* < 0.006) and the precipitation-related factor (*F* = 4.029, *R*^2^ = 0.122, *P* = 0.021) in the permutational multivariate analysis of variance.

**Fig 5:**
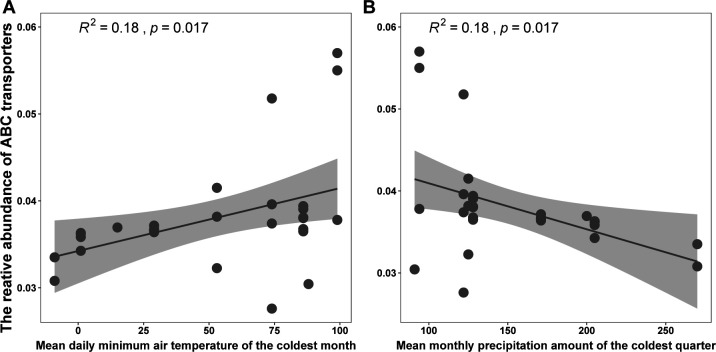
Linear regression plots between the relative abundance of ABC transporters and climatic factors. The ABC transporters showed a positive relationship with the minimum temperature of the coldest month (bio6) (**A**) and a negative relationship with the mean monthly precipitation amount of the coldest quarter (bio19) (**B**).

Many of functional items positively related to climate-related factors. For example, the relative abundance of ATP-binding cassette (ABC) transporters had an increased trend with the minimum temperature of he coldest month (bio6); ABC transporters had a decreased variation with the mean monthly precipitation amount of the coldest quarter (bio19) (Fig. 5).

**Fig 6:**
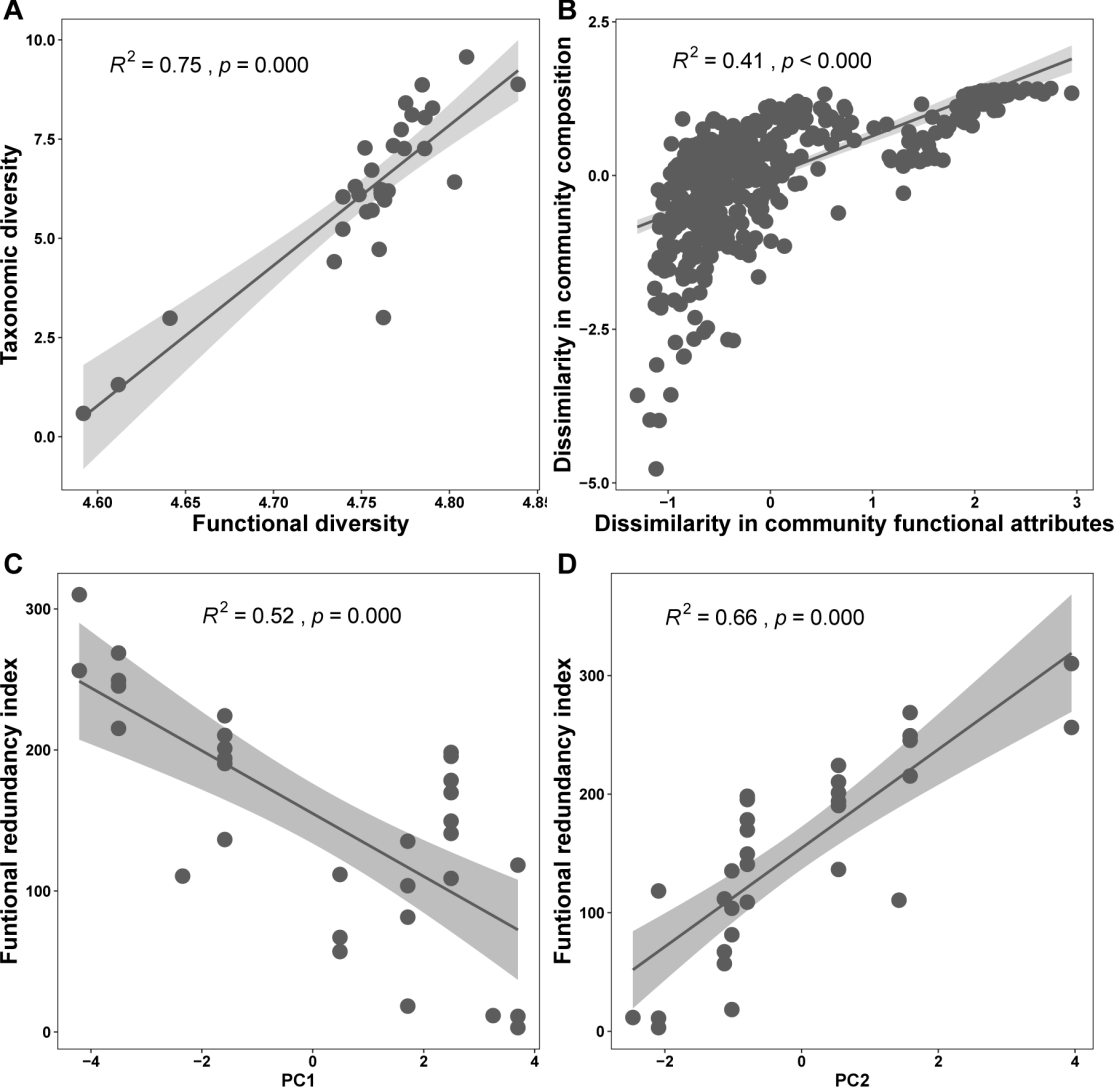
Linear regression plots of community function and microbial taxonomic diversity, and functional redundancy index and climatic factors. A linear relationship between community functional Shannon diversity and taxonomic Shannon diversity (**A**). A linear relationship between dissimilarities of microbial community composition and functional attributes (**B**). A linear relationship between microbial functional redundancy and temperature-related factor (**C**) and precipitation-related factor (**D**).

We found that skin microbial community diversity positively correlated with the community functional diversity (Shannon: *R*^2^ = 0.75, *P* < 0.001) (Fig. 6A). This pattern was also observed between microbial community compositions and functional attributes (linear regression: *R*^2^ = 0.41, *P* < 0.001; Mantel test: *r* = 0.7437, *P* < 0.001) (Fig. 6B). Furthermore, the functional redundancy index had significant correlations with the temperature and precipitation factors (Fig. 6C and D). The functional redundancy in skin microbiome increased with the minimum temperature of the coldest month (Fig. S2).

## DISCUSSION

We found that the skin microbial diversity and composition as well as community function of *P. megacephalus* frogs associate with changes of climatic conditions. The taxonomical diversity and functional diversity of the skin microbiome increased with fluctuations in climate, particularly regarding temperature variations. Furthermore, the functional attributes of the microbial community synchronously change community diversity and composition. The positive relationships between functional redundancy and climatic factors suggest that environmental filtering related to climate change exerts negative influences on microbial community functional redundancy, with a warm-wet climate could result in a lower value in microbial community functional redundancy.

The phylum Proteobacteria constituted the highest abundance in the skin microbiota harboring *P. megacephalus* frogs, as previously observed in temperate and tropical amphibian populations (28–30), and the relative abundance of this phylum positively increased with colder climate (10). The rich Proteobacteria might aid in the conversion of chitin to chitosan and cellulose (31). Despite *P. megacephalus* skin showing a site-specific microbial community structure, microbiota from certain sites were not clustered together with those from proximal sites. For instance, the microbiota from the Dongzhong (DZ) location was more similar to Dayao Mountain rather than those (Pinglong and Nakuan locations) relatively at close distance to DZ. The result suggests that geographic distances did not have a strong effect on the differences in microbiota due to the effects from local habitats (32).

We found that the alpha diversity of skin microbiome was related to climatic factors, with increased diversity along with the changing trend toward the cold and wet climates. The lower diversity of skin microbiome in warm climates might be attributed to certain microbes that have higher thermal growth optima, making them intensified competitive interactions in stable thermal environments. For instance, the genus *Acidovorax*, with its relative abundance, decreased in cold climate; this might lead to an increase in the abundance of other taxa with similar niches due to the requirements of nutrients or growth conditions (10, 33). Another important point is that certain microbes have stronger dependence on wet climate. For instance, we found the members of the families of Comamonadaceae and Moraxellaceae highly related to the precipitation factor; these families were enriched in Panama amphibians (2). Conversely, a greater microbial diversity of *P. megacephalus* in cold climate may indicate that hosts have evolved a more complex adaptive immunity (18, 20).

For skin microbial community beta diversity, our results showed that temperature-related parameters could drive the dissimilarities of the skin microbial community more than that of precipitation-related parameters. One possible explanation for this result is that temperature may strongly affect the colonization of certain microbes, leading to changes in the structure and compositions of a microbial community (34). Despite site climate difference, we identified some core taxa of the white-spotted thigh treefrog *P. megacephalus*, particularly members of the genus *Pseudomonas*, may possess vital functions for hosts, such as pathogen resistance (35, 36). The results suggest that certain skin microbiota tend to show an effective response in facing various climate conditions to facilitate harboring of hosts in a broader geographic area.

A previous study showed that microbial metabolic processes are strongly affected by natural fluctuations in temperature and precipitation (37). The identity of the metabolic features under the effects of climate shed some light on the possible mechanisms for changes in the structure and compositions of microbial communities and hosts coping with climate variations. For instance, the relative abundance of ABC transporters increased with the temperature of the coldest month and decreased with precipitation of the coldest quarter. These relationships are similar to the finding that the ABC transporters of coqui frogs’ (*Eleutherodactylus coqui*) microbiome metabolic activities were enriched during the cool-dry season (37) because the cooler climate suppresses host immunity, leading to the increased critical functions of ABC transporters, including nutrient import and molecule export (38, 39).

The community functional traits are positively related to cold climate, showing a broader distribution. This relationship is similar to the variations in microbial taxonomical diversity under climate fluctuations. Furthermore, microbial community functional attributes synchronously changed community compositions. The higher functional redundancy of skin microbiota in cooler climate suggests that *P. megacephalus* could select more cold-tolerant microbes to increase functional redundancy to facilitate community function stability of the skin microbiome. Skin microbial community composition and the distribution of functional traits are essential for amphibians to cope with the changes in climate conditions. Accordingly, further study is needed to enhance the understanding of the relationships of community functions with taxonomical diversity and climate changes by extending sampling sites for *P. megacephalus* and amphibian species.

Climate change such as higher temperature and heavier rainfall events would dramatically affect microbial diversity and function (40, 41). The temperature- and precipitation-dependent patterns of microbial community composition and function in *P. megacephalus* suggest that climate change will decrease the diversity and function stability of host skin microbiome. Importantly, the altered microbiome structure may impact host immunity and performance, even the persistence of local *P. megacephalus* populations. In addition, our study provides new insights into the compositions of core bacteria and climate-related taxa of *P. megacephalus*, contributing to the amphibian conservation through microbiota-associated strategies. However, broader geographic sampling and taxonomical sampling of natural species and populations are needed to advance the understanding of climate effects on amphibian skin microbial ecology and evolution.

In conclusion, climate influences the skin microbial community composition and functional traits in amphibian populations at different sites. Our results not only advance the understanding of the associations of climatic factors with amphibian skin microbial community diversity and community functional attributes but also contribute to predicting the adaptation of amphibians to the rapid changes in climate. We show that environmental filtering, which relates to climate, influences the diversity and composition of amphibian skin microbial and community functional attributes, as well as their relationships.

## MATERIALS AND METHODS

### Field sample collection

We collected skin swab samples from *P. megacephalus* adult individuals from nine wild sites in 2021 in Guangxi region, China. These sites included Anjiangping (*n* = 2), Cujiang (*n* = 4), Biyun Lake (*n* = 1), Tianping (*n* = 6), Dayao Mountain (*n* = 1), Dongzhong (*n* = 4), Wuzhi Mountain (*n* = 3), Nakuan (*N* = 3), Pinglong (*N* = 7). Epidermal swabs were obtained from all individuals using sterile swabs and subsequently released at their captured locations (42). We followed standardized protocols and biosecurity measures while collecting and analyzing the swabs to prevent cross contamination between individuals and transfer of pathogens across habitats.

### Bioclimatic variables

We downloaded 19 bioclimatic variables from the CHELSA v.1.2 database at a resolution of 30 arcsec (43). The details of bioclimatic variables are in Table S1. To reduce the dimensionality of climatic factors, we performed a principal component analysis. The first principal component scores represent temperature- and precipitation-related factors.

### DNA extraction and 16S rRNA gene sequencing

We extracted DNA from the swabs using Qiagen DNeasy Blood and Tissue Kit, which subsequently were amplified for genomic DNA using PCR. The V4 region of the 16S rRNA gene was amplified using specific primers (515F: GTGCCAGCMGCCGCGGTAA and 806R: GGACTACHVGGGTWTCTAAT) with barcodes. A total 15 µL of Phusion High-Fidelity PCR Master Mix (New England Biolabs), 0.2 µM of forward and reverse primers, and 10 ng DNA extract were used in each PCR amplification. The conditions for PCR amplification consisted of initial denaturation at 98°C for 1 min, 30 cycles of denaturation at 98°C for 10 s, annealing at 50°C for 30 s, and elongation at 72°C for 30 s and 72°C for 5 min. The PCR products were purified using magnetic bead purification. Sequencing libraries were generated and indexes were added. The library quality was evaluated using the Qubit v.2.0 Fluorometer (Life Technologies, California, USA) and Agilent 2100 Bioanalyzer system (Agilent Technologies, California, USA). Quantified libraries were pooled and sequenced on an Illumina platform, and 250 bp paired-end reads were generated.

### Bioinformatic analysis

The paired-end reads were processed using Python v.3.6.13 and adaptors were removed through cutadapt v.3.3. The paired-end reads were merged using FLASH v.1.2.11 (44). Quality filtering on the raw tags were performed using fastp v.0.23.1 (45). The chimera sequences were removed with the vsearch package (46).

Sequences analysis was performed through Uparse v.7.0 (47), and sequences with ≥97% similarity were assigned to the same microbial OTU. The representative sequence for each OTU was used to annotate taxonomic information using the silva database based on the Mothur algorithm. OTUs abundance was rarefied to 13,573 sequences per sample based on the sample with the least sequences. The multiple sequences were aligned using the MUSCLE v.3.8.31 (48) to analyze the phylogenetic relationship of different OTUs. Core skin microbiota was defined as OTUs that were present on at least 90% of all frog samples.

The phylogenetic investigation of communities by reconstruction of unobserved states (PICRUSt) approach can predict the KO functional profiles of microbial communities via 16S rRNA gene sequences (49). We therefore applied PICRUSt to predict the functional characteristics of microbiota which resides on *P. megacephalus* skin (50). To assess the variations in the redundancy of multiple functions of skin microbiome, we also used Tax4Fun2, which aligns 16S rRNA gene sequences to a constructed reference database to calculate the functional redundancy index (FRI) based on the species proportion and their phylogenetic relationships for a specific function (51). A higher FRI represents a higher function stability, while a lower FRI represents a higher probability of a function loss after shifts and perturbations in microbial community (51).

### Statistical analysis

The general community structure and cluster patterns of microbiome across sites were measured using the unweighted pair group with arithmetic mean method based on Bray–Curtis distances. Furthermore, to test whether climatic-related factors have strong influences on the taxonomical and community functional diversity and composition of *P. megacephalus* microbiome, we first calculated three alpha-diversity metric indices: Shannon diversity index, OTU richness, and Faith’s PD. Second, we calculated the weighted Bray–Curtis and unweighted Jacarrd distances for the microbial beta diversity and function using the adonis2 function in the vegan package with 9,999 random permutations. Principal coordinate analyses were used to visualize the distance correlations between skin microbial community and function dissimilarity in frog samples. Third, we analyzed the relationships between skin microbial alpha-diversity metrics and beta-diversity and community functional attributes between frog samples and bioclimatic variables using the Mantel test and linear regression analyses. We analyzed the changes in the microbial community composition in relation to the climatic factors at the genus level using Spearman correlation in the vegan package (52) to evaluate the significant associations of climatic factors with microbial taxa. We also determined the specific functional category in the KEGG pathways (level 3 KOs) associated with climatic factors. To test whether skin microbial community compositions and community functional attributes have similar responses to climatic changes, we analyzed the relationships between microbial community diversity and community functional attributes. Finally, we analyzed the relationships of the FRI of skin microbiome with climatic factors to explain the impact of climate variability on microbial community functional redundancy.

## Data Availability

The 16S sequence data have been uploaded to the National Center for Biotechnology Information with accession number PRJNA1155111.

## SUPPLEMENTAL MATERIAL

Supplemental Material spectrum.02358-24-s0001.pdf Table S1; Figures S1 and S2.

ASM does not own the copyrights to Supplemental Material that may be linked to, or accessed through, an article. The authors have granted ASM a non-exclusive, world-wide license to publish the Supplemental Material files. Please contact the corresponding author directly for reuse.
