# Supplementary material for: Microbiome and climate: skin microbial diversity and community functions of Polypedates megacephalus (Anura: Rhacophoridae) associated with bioclimate
Source: Microbiol Spectr. 2025 Mar 5;13(4):e02358-24. doi: 10.1128/spectrum.02358-24 (PMC11960054; doi:10.1128/spectrum.02358-24)
Supplement: Supplemental Material — Table S1; Figures S1 and S2. [file spectrum.02358-24-s0001.pdf]

Microbiome and climate: Skin microbial diversity and community functions of *Polypedates megacephalus* (Anura: Rhacophoridae) associated with bioclimate

Dan Sun[1,2], Yewei Liu[2], Shipeng Zhou[2], Madhava Meegaskumbura[2]*

[1] Guangxi Key Laboratory of Beibu Gulf Marine Biodiversity Conservation, College of Marine Sciences, Beibu Gulf University, Qinzhou 535011, People's Republic of China

[2] Guangxi Key Laboratory for Forest Ecology and Conservation, College of Forestry, Guangxi University, Nanning, Guangxi 530000, People's Republic of China

* Corresponding author email: madhava_m@mac.com

Table S1. The 19 bioclimatic variables and their units.

Name	Unit
mean annual air temperature (bio1)	°C/10
mean diurnal air temperature range (bio2)	°C/10
isothermality (bio3)	°C/10
temperature seasonality (bio4)	°C/10
mean daily maximum air temperature of the warmest month (bio5)	°C/10
mean daily minimum air temperature of the coldest month (bio6)	°C/10
annual range of air temperature (bio7)	°C/10
mean daily mean air temperatures of the wettest quarter (bio8)	°C/10
mean daily air temperatures of the driest quarter (bio9)	°C/10
mean daily mean air temperatures of the warmest quarter (bio10)	°C/10
mean daily mean air temperatures of the coldest quarter (bio11)	°C/10
annual precipitation amount (bio12)	kg m^{-2}
precipitation amount of the wettest month (bio13)	kg m^{-2}
precipitation amount of the driest month (bio14)	kg m^{-2}
precipitation seasonality (bio15)	kg m^{-2}
mean monthly precipitation amount of the wettest quarter (bio16)	kg m^{-2}
mean monthly precipitation amount of the driest quarter (bio17)	kg m^{-2}
mean monthly precipitation amount of the warmest quarter (bio18)	kg m^{-2}
mean monthly precipitation amount of the coldest quarter (bio19)	kg m^{-2}

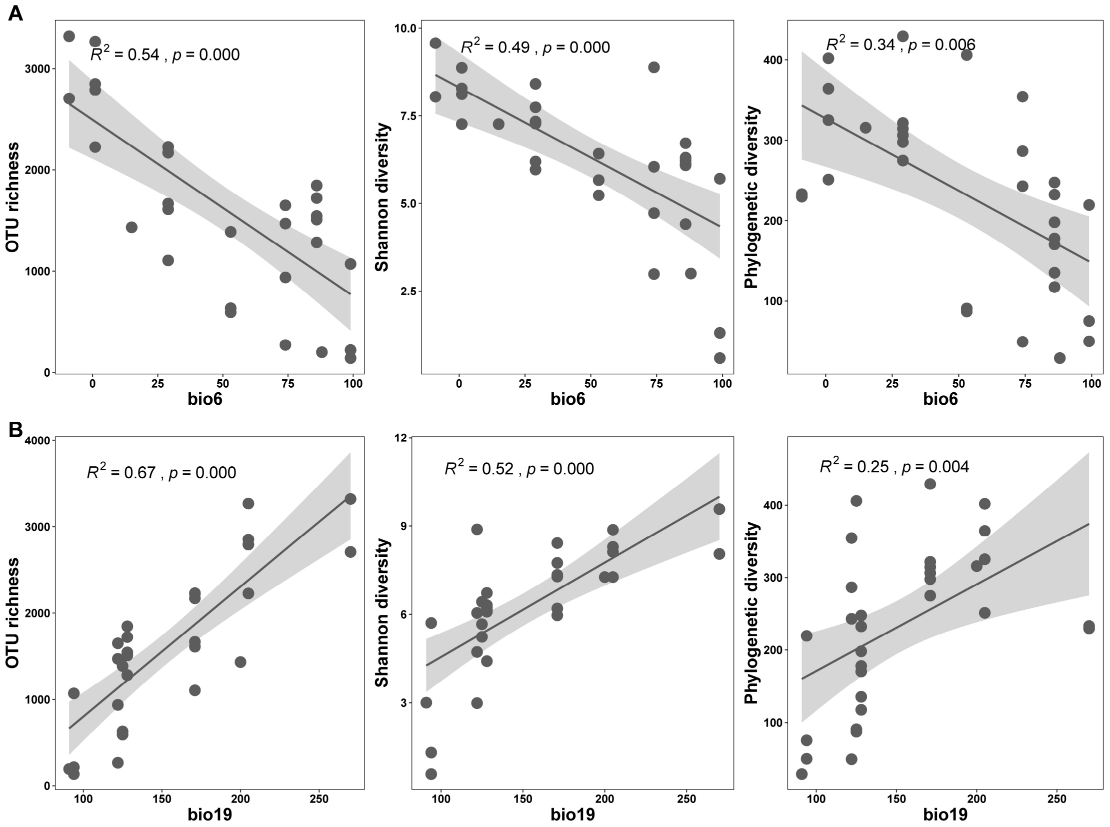

Figure S1. Linear regression plot between microbial alpha diversity and the minimum temperature of the coldest month (A) and mean monthly precipitation amount of the coldest quarter (B). Gray shading represents 95% Confidence Interval.

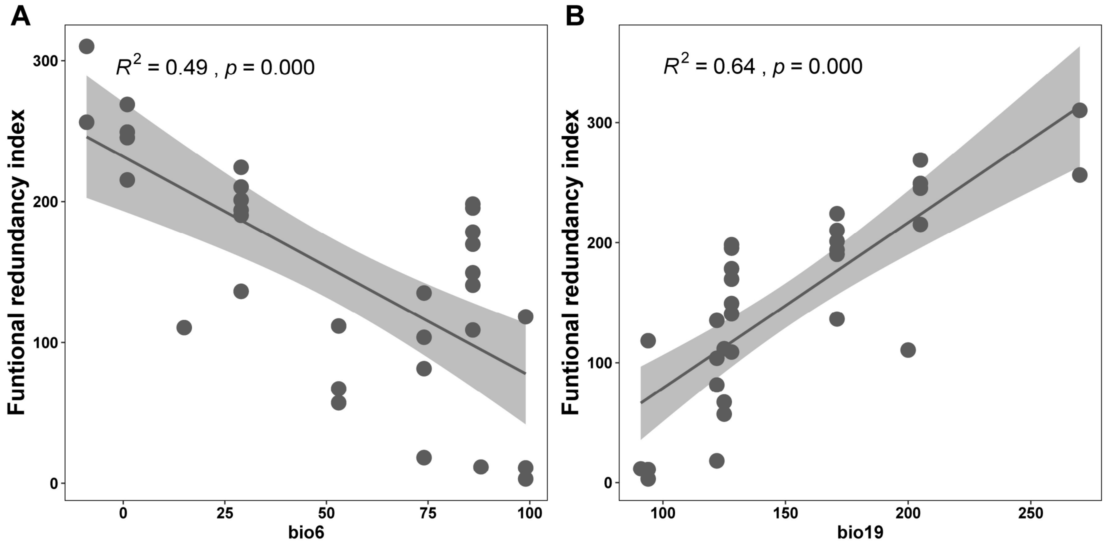

Figure S2. Linear regression plot between community functional redundancy index and the minimum temperature of the coldest month (A) and mean monthly precipitation amount of the coldest quarter (B). Gray shading represents 95% Confidence Interval.
